# 2-DG Regulates Immune Imbalance on the Titanium Surface after Debridement

**DOI:** 10.3390/ijms241411431

**Published:** 2023-07-13

**Authors:** Xingchen Liu, Shudan Deng, Jiaxin Xie, Chunxin Xu, Zhuwei Huang, Baoxin Huang, Zhuofan Chen, Shoucheng Chen

**Affiliations:** 1 Hospital of Stomatology, Sun Yat-sen University, Guangzhou 510055, China; 2 Guangdong Provincial Key Laboratory of Stomatology, Guangzhou 510055, China; 3 Guanghua School of Stomatology, Sun Yat-sen University, Guangzhou 510055, China; 4 Guangdong Research Center for Dental and Cranial Rehabilitation and Material Engineering, Guangzhou 510055, China

**Keywords:** peri-implantitis, osteoimmunomodulation, immune imbalance, osteogenesis, bone regeneration, clinical treatments

## Abstract

Peri-implantitis requires clinical treatments comprised of mechanical and chemical debridement to remove bacterial biofilms. Bone regeneration on the titanium surface after debridement has been a topical issue of peri-implantitis treatments. Increasing evidence has revealed that the immune microenvironment plays a key role in regulating the bone regeneration process. However, it remains unclear what kind of immune microenvironment the titanium surface induces after debridement. In the study, model titanium surface after debridement was prepared via biofilm induction and mechanical and chemical debridement in vitro. Then, the macrophages and naïve CD4^+^ T lymphocytes were cultured on the titanium surface after debridement for immune microenvironment evaluation, with the original titanium surface as the control. Next, to regulate the immune microenvironment, 2-DG, a glycolysis inhibitor, was further incorporated to regulate macrophages and CD4^+^ T lymphocytes at the same time. Surface characterization results showed that the bacterial biofilms were completely removed, while the micro-morphology of titanium surface altered after debridement, and the element composition did not change. Compared with the original titanium disc, titanium surface after debridement can lead to the inflammatory differentiation of macrophages and CD4^+^ T lymphocytes. The percentage of M1 and Th17 inflammatory cells and the expression of their inflammatory factor genes are upregulated. However, 0.3 mmol of 2-DG can significantly reduce the inflammatory differentiation of both macrophages and CD4^+^ T lymphocytes and inhibit their expression of inflammatory genes. In conclusion, although bacterial biofilms were removed from titanium surface after debridement, the surface topography changes could still induce immune imbalance and form an inflammatory immune microenvironment. However, this inflammatory immune microenvironment can be effectively reversed by 2-DG in vitro, thus creating an immune microenvironment conducive to osteogenesis, which might provide a new perspective for future therapy of peri-implantitis.

## 1. Introduction

Peri-implantitis is a prevalent and serious disease that compromises the health of dental implants. With the increasing applications of dental implants, there has been a rapid rise of peri-implantitis patients. A cross-sectional study reported the prevalence of peri-implantitis was approximately 35%, which could increase to 62.1% among implants with plaque [1]. Similarly, Krebs et al. reported that peri-implantitis occurs in an estimated 7.9% to 15.0% of patients with dental implants after 18.9 years follow-up [2]. The high prevalence of peri-implantitis underscores the urgent need for effective treatment strategies to address this escalating health challenge [3,4,5,6].

Currently, the standard approaches of peri-implantitis treatments involve the use of mechanical and chemical debridement, guided bone regeneration, and maintenance therapy [7,8,9]. These series approaches remove bacterial biofilm and inflammatory tissue from the implant surface; however, recent research indicates that such treatments inevitably modify the original surface of the implant, affecting surface characteristics such as roughness, topography, wettability, etc. [10,11]. Bone regeneration and osteointegration on the altered titanium surface after debridement has been a topical issue of peri-implantitis treatments.

Bone regeneration and integration around implants is a multifaceted process that involves the participation of several cell types from distinct systems. With the progression of osteoimmunomodulation research, increasing evidence has revealed the significance of the local immune microenvironment in regulating bone formation and integration [12,13,14]. It has been observed that modulating macrophage polarization towards M2 and promoting T lymphocyte differentiation towards regulatory T cells (Treg) and T helper 2 (Th2) can effectively inhibit inflammation and facilitate bone integration. On the other hand, an adverse immune microenvironment, characterized by macrophage polarization towards the M1 phenotype and differentiation of T cells towards Th17 and Th1, can lead to tissue damage, hinder osteogenic differentiation, and disrupt the balance of bone remodeling, ultimately resulting in delayed or failed bone integration [15,16,17,18]. However, it remains unclear what immune microenvironment the titanium surface induces after debridement.

2-Deoxyglucose (2-DG) has been reported to be extensively utilized in cancer therapy owing to its potential to suppress the glycolysis of tumor cells and, thus, inhibit tumor progression [19,20,21,22]. Moreover, research has shown that almost all pro-inflammatory immune cells, including innate and adaptive immunity primarily rely on glycolysis as their main glucose metabolic pathway [23,24,25,26]. It has been proven that a certain concentration of 2-DG can target cellular glycolysis and respectively inhibit the pro-inflammatory differentiation of immune cells such as Th17/Treg and M1/M2 [24,27,28,29], indicating the high potential of 2-DG for overall regulation of immune microenvironments around dental implants.

The aim of this study is to first evaluate the immune microenvironment of the titanium surface after debridement. The evaluation of the immune microenvironment includes both innate immunity (macrophage) and adaptive immunity (CD4^+^ T lymphocyte). When we discovered that an immune microenvironment with inflammatory imbalance was induced by the titanium surface after debridement, we further explored the potential for 2-DG to simultaneously modulate macrophage and CD4^+^ T lymphocyte immune imbalance, with the goal of establishing an immune microenvironment both in innate and adaptive immunity, which is conducive to osteogenesis.

## 2. Results

### 2.1. Construction and Characterization of the Titanium Surface after Debridement

**Figure 1:**
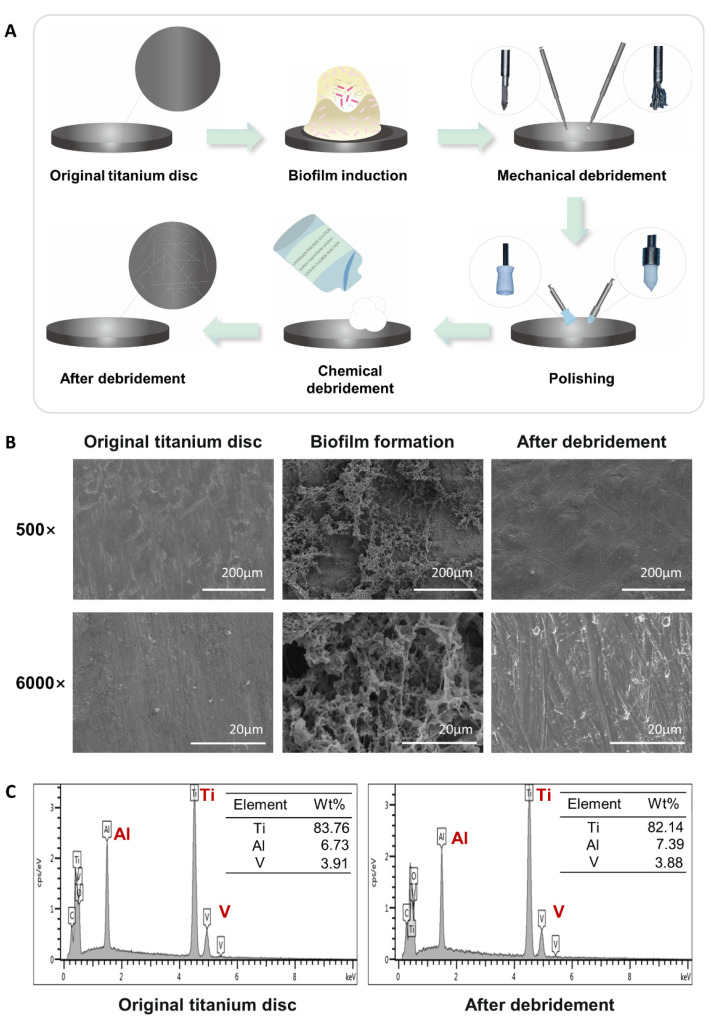
Titanium surface after debridement. (**A**) Flow chart: construction of the titanium surface after debridement in vitro. (**B**) SEM of the titanium surface. (**C**) EDS of the original titanium surface and the titanium surface after debridement.

To simulate titanium surfaces on peri-implantitis following debridement in vitro, we developed an in vitro simulation method (Figure 1A). First, Streptococcus mutans, a major pathogenic bacterium associated with peri-implantitis and an early colonizer of oral biofilm [30,31,32,33,34,35], was inoculated on medical-grade TC4 titanium discs. To simulate the standard clinical approaches [36,37,38] for treating peri-implantitis, we subsequently subjected the bacterial-colonized titanium surface to mechanical debridement, polishing, and chemical debridement. The titanium disc surface morphology was observed by SEM (Figure 1B), and the elemental composition was evaluated by EDS analysis (Figure 1C). We found that although a series of treatments effectively removed the bacterial biofilm on the titanium surface, it inevitably caused significant changes to the original surface morphology. SEM results showed that after mechanical debridement, polishing, and chemical debridement, the titanium disc surface produced more scratches, altering the surface roughness. At a magnification of 6000 times of SEM, irreversible changes in the microstructure of the titanium surface were observed (Figure 1B). To determine whether the elemental composition of the titanium disc had changed, EDS analysis was performed on the surface before and after biofilm contamination and debridement, and we found that the elemental composition of the titanium discs mainly remained titanium, aluminum, and vanadium, and the composition and proportion of the elements did not change.

### 2.2. Titanium Surface after Debridement Results in Immune Imbalance

**Figure 2:**
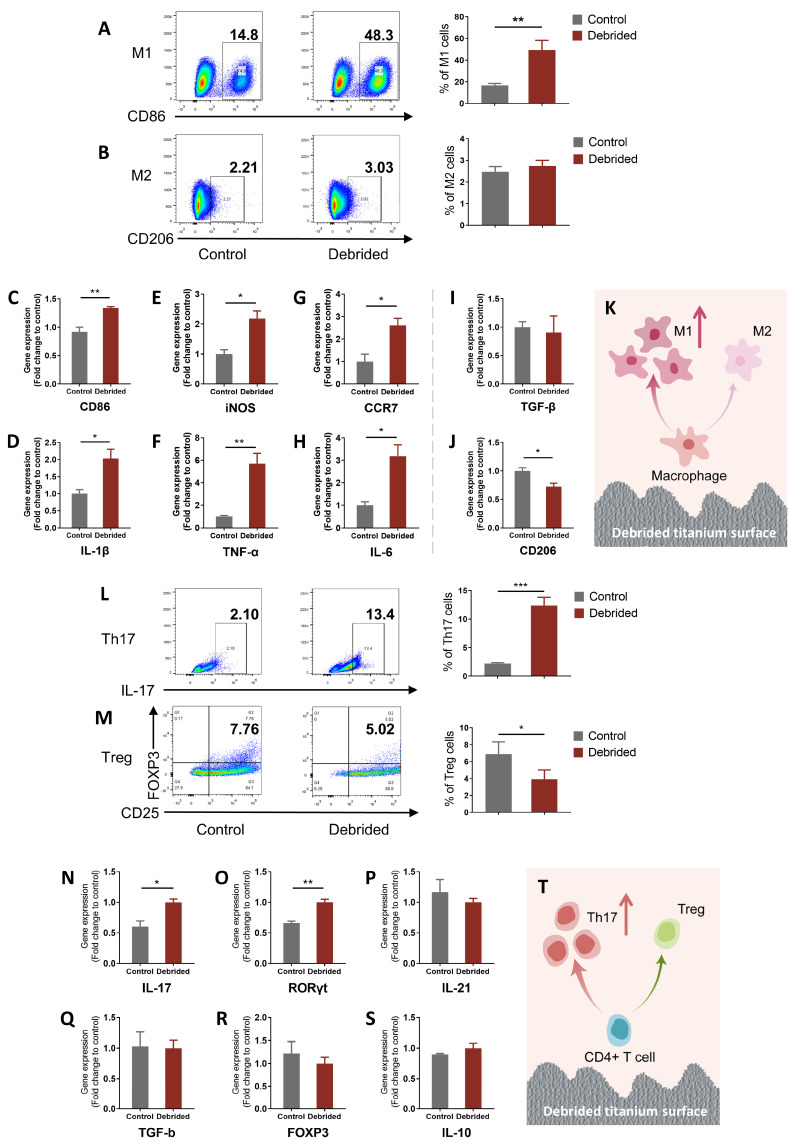
Titanium surface after debridement results in immune imbalance. (**A**,**B**) Flow cytometry of macrophages. (**C**–**J**) qRT-PCR of macrophages. (**K**) Model Diagram: titanium surface after debridement results in increased M1 percentage. (**L**,**M**) Flow cytometry of naïve CD4^+^ T cells. (**N**–**S**) qRT-PCR of T cells. (**T**) Model Diagram: titanium surface after debridement results in increased Th17 percentage. (Control: cells incubated on the original titanium disc; debrided: cells incubated on the titanium disc after debridement). * *p* < 0.05; ** *p* < 0.01; *** *p* < 0.001.

To clarify the effect of titanium surface after debridement on immune cell differentiation, M0 macrophages and naïve CD4^+^T cells were co-cultured respectively with the debrided titanium discs and measured by flow cytometry and qRT-PCR. Through flow cytometry, we found that the debrided titanium surface induced an immune imbalance in macrophages and naïve CD4^+^ T cells and facilitated their differentiation towards a pro-inflammatory phenotype. The percentage of M1 in the debrided group (cells on titanium discs after debridement) was nearly three times higher than that in the control group (cells on original titanium discs), which was significantly different, bringing about the increasing ratio of M1/M2 from 6.76 to 18.01 (Figure 2A,B). The expression of M1-related genes in the debrided group, such as CD86, iNOS, CCR7, IL-1β, TNF-α, and IL-6, was significantly upregulated. However, the expression of M2-related genes had no statistical difference (TGF-β) or was slightly downregulated (CD206) from controls (Figure 2C–J). Similarly, flow cytometry results also showed that the significantly increased percentage of Th17 and decreased percentage of Treg resulted in the increasing ratio of Th17/Treg in the debrided group from 0.32 to 3.15 (Figure 2L,M). The qRT-PCR results showed that Th17-related genes, such as IL-17, RORγT, and IL-21, were also upregulated, and Treg-related genes, such as TGF-b, FOXP3, and IL-10, had no statistical change (Figure 2N–S). The results of flow cytometry and qRT-PCR confirm each other.

### 2.3. 2-DG Could Regulate the Pro-Inflammatory Immune Imbalance of Macrophages Induced by Titanium Surface after Debridement

**Figure 3:**
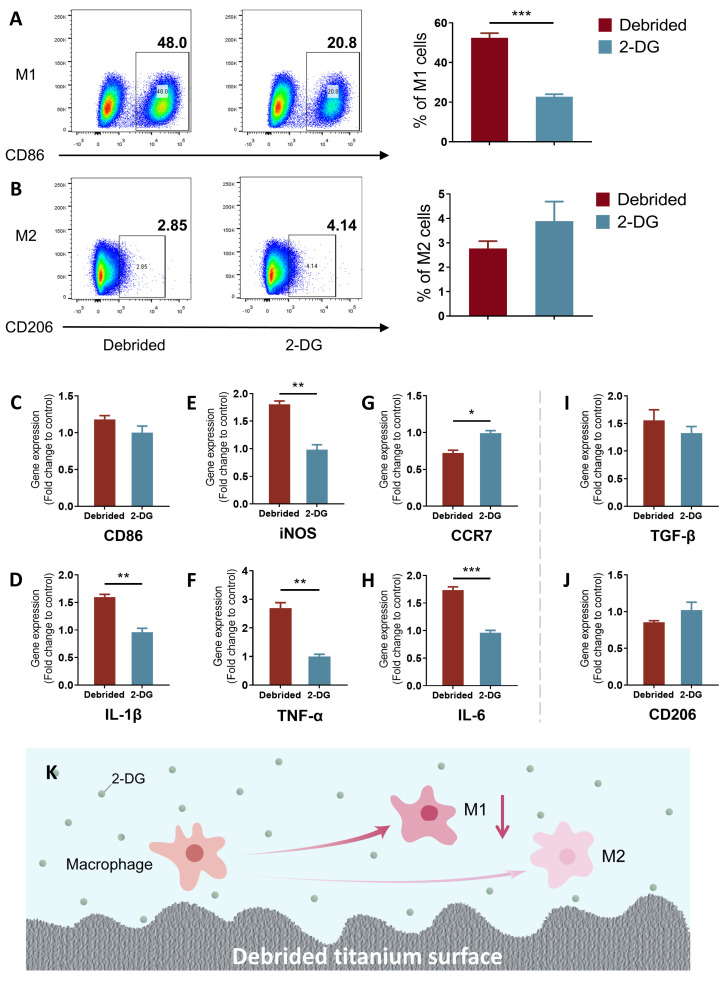
2-DG could regulate the pro-inflammatory immune imbalance of macrophages induced by titanium surface after debridement. (**A**,**B**) Flow cytometry of debrided group and 2-DG group. (**C**–**J**) qRT-PCR of debrided group and 2-DG group. (**K**) Model diagram: 2-DG could reverse the increased percentage of M1 macrophage induced by the titanium surface after debridement. (debrided group: cells incubated on the titanium disc after debridement; 2-DG group: cells incubated with 2-DG on the titanium disc after debridement). * *p* < 0.05; ** *p* < 0.01; *** *p* < 0.001.

To assess the regulatory effect of 2-DG on pro-inflammatory immune imbalance of macrophages induced by titanium surface after debridement, M0 macrophages were co-cultured with titanium discs after debridement in medium with/without 2-DG. The results of flow cytometry showed, under the presence of 2-DG (cells incubated with 2-DG on titanium discs after debridement), the percentage of M1 macrophages decreased significantly compared with the debrided group (cells incubated on titanium discs after debridement), while the percentage of M2 had no statistical change, which reduced the ratio of M1/M2 from 18.94 to 5.85 (Figure 3A,B and Figure S2). Moreover, qRT-PCR results showed that M1-related genes (CD86, iNOS, CCR7, IL-1β, TNF-α, and IL-6) in the 2-DG group were mostly downregulated, and M2-related gene (TGF-β and CD206) expression had no statistical difference instead (Figure 3C–J). Therefore, it can be concluded that 2-DG, to a certain extent, reversed the immune imbalance of M1/M2 induced by the titanium surface after debridement and inhibited pro-inflammatory differentiation of macrophages.

### 2.4. 2-DG Could Regulate the Pro-Inflammatory Immune Imbalance of T Cells Induced by Titanium Surface after Debridement

**Figure 4:**
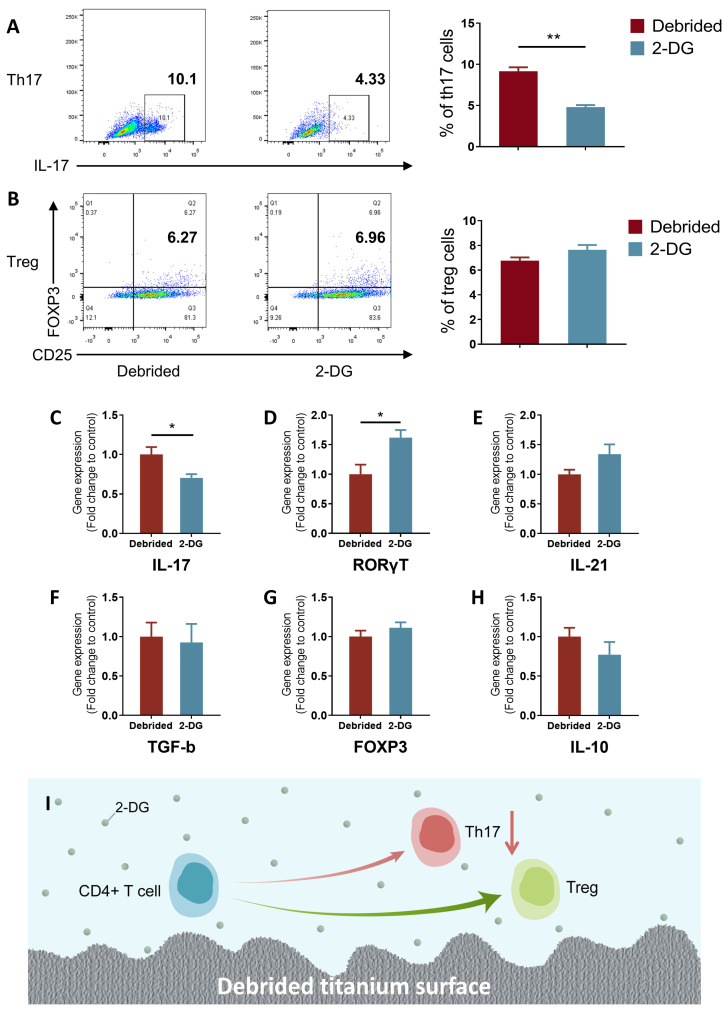
2-DG could regulate the pro-inflammatory immune imbalance of naïve CD4^+^ T cells induced by titanium surface after debridement. (**A**,**B**) Flow cytometry of debrided group and 2-DG group. (**C**–**H**) qRT-PCR of debrided group and 2-DG group. (**I**) Model diagram: 2-DG could reverse the increased percentage of Th17 cells induced by titanium surface after debridement. (debrided group: cells incubated on the titanium disc after debridement; 2-DG group: cells incubated with 2-DG on the titanium disc after debridement). * *p* < 0.05; ** *p* < 0.01.

To assess the regulatory effect of 2-DG on pro-inflammatory immune imbalance of naïve CD4^+^ T cells induced by titanium surface after debridement, naive CD4^+^ T cells and titanium discs after debridement were co-cultured in medium with/without 2-DG. Similar to macrophages, the results of flow cytometry showed that the percentage of Th17 in the 2-DG group (cells incubated with 2-DG on titanium discs after debridement) significantly decreased, and the percentage of Treg had no statistical change (Figure 4A,B). The ratio of Th17/Treg was reduced over two times from 1.35 to 0.63. According to the qRT-PCR results of Th17-related gene, although the expression of RORγT was increased after addition of 2-DG, IL-17, the most important inflammatory factor produced by th17 cells, was significantly decreased, and there were no changes in Treg-related genes, such as TGF-b, FOXP3, and IL-10 (Figure 4C–H). These data together indicated that 2-DG might, to some extent, reverse the pro-inflammatory differentiation trend of Th17/Treg induced by titanium surface after debridement and inhibit pro-inflammatory differentiation of naïve CD4^+^ T cells.

## 3. Discussion

The increasing prevalence of dental implants has made peri-implantitis a pressing issue, prompting a need for effective treatment strategies. Currently, mechanical debridement and chemical irrigation are the primary therapeutic options for peri-implantitis. Although these methods remove bacterial biofilm and inflammatory tissue from the implant surface, they are suboptimal in terms of the long-term osteogenic efficacy of the implant [39,40]. Current research in this area predominantly focuses on the direct osteogenic potential of the debrided implant, while bone regeneration and integration are governed by multiple regulatory systems, including the immune microenvironment [12,41,42]. This study highlights the possibility that even after debridement, the implant may still lead to imbalances in the immune microenvironment. Achieving successful bone regeneration and integration requires coordinated regulation of multiple regulatory systems, including the immune response. Hence, it is crucial to consider the immune microenvironment as a critical determinant of implant outcomes. Moreover, we investigate the potential of 2-deoxy-D-glucose (2-DG) to comprehensively regulate innate and adaptive immunity, with the aim of establishing an immune microenvironment that supports osteogenesis.

Original implants promote osseointegration through their surface morphology, wettability, and hydrophilicity, among other characteristics. In the case of treatment of peri-implantitis, the bone re-integration takes place at the surface following debridement. Research has shown that mechanical debridement and chemical treatments inevitably modify the original surface of the implant, including roughness, topography, wettability, and other factors. Metal instruments with high mechanical strength can significantly damage the surface morphology of implants, leading to a noticeable reduction in surface roughness and biocompatibility. On the other hand, instruments made of plastics and rubber, which have lower strength compared to the implants, can cause scratches on the implant surface, resulting in the attachment and deposition of debris. Ignoring the changes in surface characteristics of dental implants can have a detrimental impact on the efficacy of traditional treatment strategies, leading to adverse effects on implant–bone integration [10,11,43]. Consistent with previous studies, the series of treatments resulted in irreversible changes in the microstructure of the titanium surface (Figure 1A,B).

It is widely accepted that after debridement, the surface of the implant should induce a favorable immune microenvironment for bacterial biofilms and inflammatory tissue to be removed [7,8]. However, our research has revealed that debridement-induced changes on the surface of titanium can still result in an immune imbalance characterized by the pro-inflammatory differentiation of immune cells (Figure 2K,T). Specifically, the debridement may change the morphology of the titanium surface, which further activates macrophages and CD4^+^ T lymphocytes, prompting their differentiation into the M1 and Th17 phenotypes that produce pro-inflammatory cytokines and other mediators. These pro-inflammatory cells and cytokines can contribute to local inflammation in the bone tissue, impacting bone healing and regeneration and hindering bone integration [12,16,44,45].

**Figure 5:**
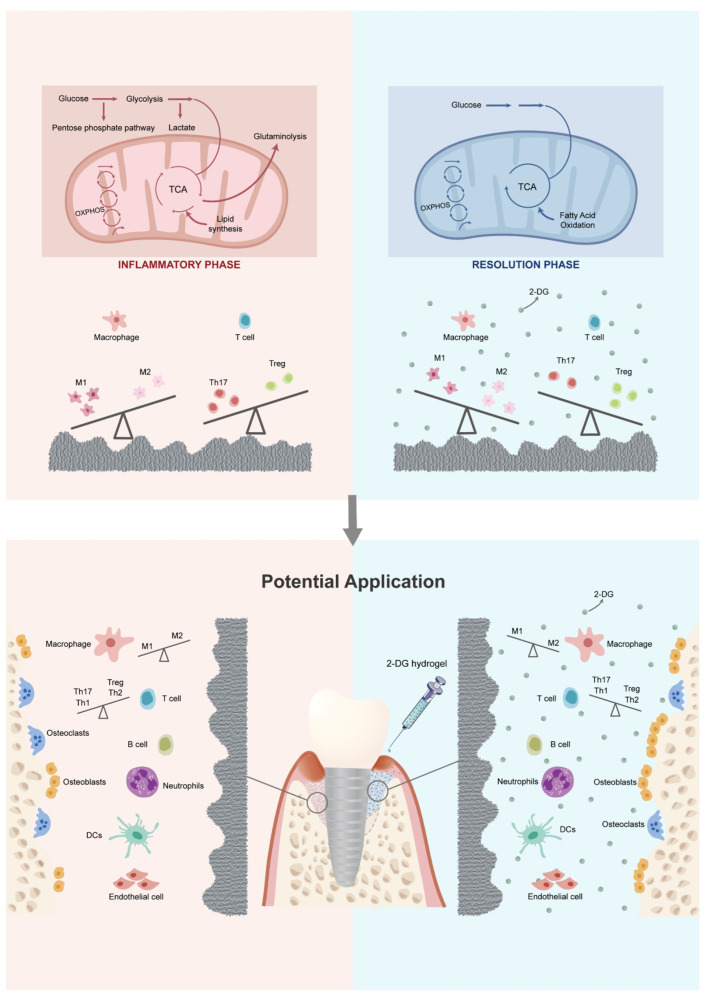
Mechanism and regulatory effect of 2-DG on immune cells and potential application of 2-DG in peri-implantitis.

2-DG has been reported to be widely used in cancer treatment due to its ability to inhibit tumor progression by targeting and suppressing the glycolysis of tumor cells [19,20]. Meanwhile, research has shown that almost all pro-inflammatory immune cells in both innate and adaptive immunity primarily rely on glycolysis as their main metabolic pathway [29]. In previous studies, it has been consistently reported that the concentration of 2-DG used can have varying effects on lymphocyte proliferation and T cell differentiation into Th17 cells. High concentrations of 2-DG, typically ranging from 10 to 50 mM, have been shown to negatively impact lymphocyte proliferation. Conversely, lower concentrations of 2-DG, specifically 0.5 mM and 1 mM, have been found to have no significant effect on lymphocyte proliferation and, interestingly, can even inhibit the differentiation of T cells into Th17 cells, which are known to contribute to inflammatory responses [46,47]. Furthermore, there is evidence indicating that 2-DG concentrations below 0.3 mM can specifically target macrophage polarization. Studies have demonstrated that at these lower concentrations, 2-DG can effectively inhibit the polarization of macrophages towards the pro-inflammatory M1 phenotype while not affecting the alternative M2 polarization state. This suggests that 2-DG has the potential to modulate the immune response by selectively targeting and suppressing the M1 polarization of macrophages, which are involved in the promotion of inflammatory processes [24]. Taking these findings into consideration, it becomes evident that the concentration of 2-DG used in experimental settings plays a crucial role in determining its effects on different immune cell populations. The choice of concentration should be carefully considered, taking into account the specific objectives of the study and the desired immune response modulation. Findings from this study demonstrate that the use of a specific concentration of 2-DG (0.3 mM) can simultaneously rectify both the innate and adaptive pro-inflammatory immune imbalance induced by the titanium surface after debridement (Figure 3 and Figure 4). As a result, the application of 2-DG may be able to holistically regulate the imbalanced immune microenvironment by targeting cell glycolysis, establishing an environment that is more favorable for bone formation and less conducive to bone resorption (Figure 5). The ability of 2-DG to regulate immune responses holds promise for its application in the future treatment of peri-implantitis. By rectifying the immune imbalance induced by the titanium surface, 2-DG has the potential to promote a more conducive environment for successful bone formation and integration, ultimately improving the long-term outcomes of implant treatments.

However, it should be noted that the titanium disc used in the experiment may not fully represent the characteristics of clinically used dental implants. In addition, the immune microenvironments induced by the titanium surface after debridement are complicated and far from well understood. The responses of other immune cells (neutrophils, B cells, dendritic cells, mast cells, etc.) on the debrided titanium surface and the influence of one immune cell type on the other should be thoroughly investigated in the future. In addition, adding a group of mechanically and chemically treated titanium discs without biofilm formation could provide a valuable baseline comparison and help differentiate the effects of biofilm formation from the effects of mechanical and chemical treatments alone. Currently, due to the lack of specific 2-DG inhibitors [20,48], the exact influence of 2-DG on macrophages and T cells remains to be specifically elucidated. Further research is warranted to fully elucidate the mechanisms by which 2-DG modulates the immune response and its specific effects on bone regeneration and integration. Additionally, the optimal dosage, administration route, and potential side effects of 2-DG need to be explored to ensure its safe and effective use in clinical settings. At the same time, further evaluation of osteogenic efficacy is also needed through additional experiments in vitro and in vivo.

Another limit of this study is the lack of investigation into the specific molecular or structural cues that contribute to immune imbalance following debridement. While our study focused on evaluating the effects of biofilm debridement on immune balance, the precise mechanisms and cues driving these changes remain to be fully elucidated. Understanding the molecular and structural cues involved in immune imbalance after debridement is crucial for developing targeted therapeutic strategies and improving clinical outcomes. Further studies are warranted to investigate the biological analyses, specific molecules, and structure and tissue microenvironment alterations that contribute to immune imbalance after debridement. Techniques such as high-throughput omics analyses, advanced imaging modalities, and in-depth molecular profiling could provide valuable insights into the underlying molecular and structural cues involved. Additionally, incorporating in vivo models and clinical samples from patients undergoing debridement procedures may offer a more comprehensive understanding of the immune response and potential factors contributing to immune imbalance.

In conclusion, although bacterial biofilms were removed from the titanium surface after debridement, the surface topography changes could still induce both the innate and adaptive immune imbalance and form an inflammatory immune microenvironment. However, this inflammatory immune microenvironment can be effectively reversed by 2-DG in vitro, thus creating an immune microenvironment conducive to osteogenesis, which might provide a new perspective for future therapy of peri-implantitis.

## 4. Materials and Methods

### 4.1. Titanium Discs

For the experiments, sterile medical TC4 (Ti-6Al-4V) discs, with diameter of 30 mm and thickness of 1 mm in size (provided by Sente Material Technology Co., Ltd., Taizhou, China), were used.

### 4.2. Bacterial Culture and Biofilm Formation

Streptococcus mutans strain UA159 (ATCC 700610), a Gram-positive bacterium known to be involved in the early stage of biofilm formation, was used for the study. For routine use, *S. mutans* was subcultured on brain heart infusion (BHI) agar (Sigma-Aldrich, St. Louis, MO, USA). The sterile titanium disc samples for each group were fixed on a 6-well plate. *S. mutans* was inoculated in BHI broth and then incubated for 16 h at 37 °C in an orbital shaker incubator at 50 rpm. The cultures were then centrifuged at 5000× *g* rpm for 5 min at 4 °C. Thereafter, the pellets were resuspended in 1 ml of new medium with optical density (OD) adjusted from 0.5 to 0.6 at a wavelength of 600 nm. Each experimental titanium disc sample was inoculated with *S. mutans* (1.0 × 10^7^ CFU/mL) and cultured for 24 h [30,41,49,50].

### 4.3. Debridement on Bacterial-Colonized Titanium Discs

All treatments were performed by a single researcher in the study. The bacterial-colonized titanium discs were first mechanically debrided using two different NiTi brushes in a sequence (NiTi brush, Nano and Pocket, HANS Korea, Seoul, Republic of Korea). The debridement was performed at a speed of 800 rpm for 40 s with each brush sequentially. The titanium discs were further polished successively using a nylon brush and rubber cups on the disc surfaces. Polishing was carried out successively for 30 s at a speed of 500 rpm (Supplementary Materials Figure S1). The operator ensured to use the lowest feasible lateral pressure while performing instrumentation. After mechanical debridement and polishing, the titanium discs underwent chemical debridement using specific agents. The chemical debridement involved 15 back-and-forth strokes using cotton pellets soaked in different chemical solutions. These solutions included a 3% hydrogen peroxide solution (H_2_O_2_; Hengjian Pharmaceutical Co., Guangzhou, China), a 5.25% sodium hypochlorite solution (NaClO; Jiulong Biological Ltd., Guangzhou, China), and a 0.9% sodium chloride solution (NaCl; Sigma-Aldrich Co., St. Louis, MO, USA). A control group was included, consisting of original titanium discs without bacterial colonization and without any treatments. The procedures were performed meticulously to maintain consistency and ensure standardization across all treated discs. The use of different brushes, polishing tools, and chemical agents aimed to achieve effective mechanical and chemical debridement of the bacterial-colonized titanium discs. These treatments were performed in a controlled manner to mimic clinical debridement procedures and evaluate their effects on the titanium disc surfaces.

### 4.4. Scanning Electron Microscopy (SEM) and Chemical Analyses

After the titanium discs were subjected to the various treatments for 24 h, the titanium disc surface was observed by scanning electron microscopy (SEM). Images at high magnification were taken at different locations on each disc by SEM (JSM-7600F, JEOL, Brno, Tokyo, Japan). The surface of 1 disk sample per group was analyzed using energy-dispersive X-ray spectroscopy (EDS; JSM-7600F, JEOL, Brno, Tokyo, Japan).

### 4.5. Cell Preparation and Culture

Raw264.7 cells were obtained from Zhong Qiao Xin Zhou Biotechnology Co., Ltd. (Shanghai, China). Cells were cultured in Dulbecco’s Modified Eagle’s Medium (DMEM, Gibco, New York, NY, USA) containing 10% fetal bovine serum (Themofisher, Waltham, MA, USA) and 1% antibiotics (Gibco, New York, NY, USA). Cells were grown in a cell incubator at 37 °C with 5% CO_2_ and passaged every three days or when confluent. Cells were transferred into a 6-well plate and cultured on the titanium discs in normal Raw264.7 cells medium with/without 2-DG (0.3 mmol, cat. HY-13966, MCE, Shanghai, China) for another 1 day [24,51].

Lymphocytes were isolated from the spleens of 4-week male SD rats. Single cell suspensions were obtained by meshing the spleens through a 70 μm cell strainer, and lymphocytes were obtained using a lymphocyte separation solution (DAKEWE, Shenzhen, China). Naïve CD4^+^ T cells were extracted using the EasySep™ Rat T Cell Isolation Kit (Stemcell, Vancouver, BC, Canada) according to the manufacturer’s protocols. Purity of each fraction was >90%, with >98% viability. Naïve CD4^+^ T cells were activated with plate-bound anti-CD3 (2 µg/mL) and soluble anti-CD28 (1 µg/mL) antibodies in RPMI 1640 medium (Gibco, New York, NY, USA) containig 10% fetal bovine serum (Themofisher, Waltham, MA, USA), 2 mM L-glutamine (Biological Industries, Kibbutz Beit Haemek, Israel), and 1% antibiotics (Gibco, New York, NY, USA) for 24 h. Then, cells were transferred into a new 6-well plate and cultured on the titanium discs in normal T cell medium with/without 2-DG (0.3 mmol) for another 1 day [52,53].

### 4.6. Flow Cytometry

Cell differentiation trends were quantified using flow cytometry. For flow cytometry, the cells (1.0 × 10^6^ per sample) were washed with PBS and incubated with antibody in a blocking solution (1% *v*/*v* BSA in PBS) for 30 min. RAW264.7 cells were stained with APC-conjugated anti-mouse CD86 antibody (cat.105011, Biolegend, San Diego, CA, USA) or PE-conjugated anti-mouse CD206 antibody (cat.141705, Biolegend, San Diego, CA, USA) to analyze the Macrophage M1 or M2 frequency, respectively. For Th17 cell analysis, T cells were stained with FITC-conjugated anti-rat CD4 antibody (cat.201505, Biogend, San Diego, CA, USA) and eFluor^TM^506 anti-rat IL-17A antibody (cat.69-7177-82, ThermoFisher, Waltham, MA, USA) and stained with PE-conjugated anti-rat CD25 antibody (cat.202105, Biolegend, San Diego, CA, USA) and Alexa Fluor^®^ 488 anti-rat Foxp3 antibody (cat.320012, Biolegend, San Diego, CA, USA) to analyze Treg cell. Data were analyzed with FlowJo_V×10.8 software (FlowJo LLC, Ashland, OR, USA).

### 4.7. RNA Extraction and qRT-PCR

The total RNA was extracted using RNA quick purification kit (ES science, Beijing, China). The extracted total RNA was measured by Nanodrop (Thermo Fisher, Waltham, MA, USA) and then converted to complementary DNA by Hieff^®^ Ⅲ 1st strand cDNA Synthesis Kit (Yeasen, Shanghai, China). Then, cDNA samples were diluted and used for RT-qPCR analysis on an ABI two-step system (Applied Biosystems, Waltham, MA, USA) using Hieff^®^ qPCR SYBR Green Master Mix (Yeasen, Shanghai, China). Primer sequences are shown in Supplementary Materials (Supplementary Materials Table S1). Samples were calculated based on the 2^−ΔΔCt^ method using GAPDH as a reference gene.

### 4.8. Statistic Analysis

All statistical analyses were performed using GraphPad Prism 8.02 software (San Diego, CA, USA). Differences between groups were determined using unpaired *t*-test. Statistical significance was set at 0.05 (α = 0.05). The significance between groups is marked as follows: * (*p* < 0.05), ** (*p* < 0.01), and *** (*p* < 0.001).

## Data Availability

The data that support the findings of this study are available from the corresponding authors upon reasonable request.

## Supplementary Materials

The following supporting information can be downloaded at: https://www.mdpi.com/article/10.3390/ijms241411431/s1.
