# Supplementary material for: 2-DG Regulates Immune Imbalance on the Titanium Surface after Debridement
Source: Int J Mol Sci. 2023 Jul 13;24(14):11431. doi: 10.3390/ijms241411431 (PMC10380309; doi:10.3390/ijms241411431)
Supplement: Supplementary file 1 [file ijms-24-11431-s001.zip › ijms-2369605-supplementary.pdf]

2-DG regulates immune imbalance on the titanium surface after debridement

X. Liu[1,], S. Deng[1,*], J. Xie[1], C. Xu[1], Z. Huang[1], B. Huang[1], Z. Chen[1] and S. Chen[1]*

[1] Hospital of Stomatology, Guanghua School of Stomatology, Sun Yat-sen University and Guangdong Provincial Key Laboratory of Stomatology and Guangdong Research Center for Dental and Cranial Rehabilitation and Material Engineering, Guangzhou, 510055, China

*The authors contributed equally

Correspondence:

chenshch8@mail.sysu.edu.cn (Dr. Shoucheng Chen)

chzhuof@mail.sysu.edu.cn (Prof. Zhuofan Chen)

Supplementary Table S1. The primers used for quantitative PCR.

Gene	Sequence
mouse-GAPDH	Forward 5'-TCAGTCAGCAATGCCTCCTGCAC-3'
	Reverse 5'-TCTGTCTGGGTGGCAGTGATGGC-3'
mouse-CD86	Forward 5'-CTGCCTGCTCATCATTGTATGTCAC-3'
	Reverse 5'-ACTGACTGCCTTCACTCTGCATTTG-3'
mouse-iNOS	Forward 5'-CAGACAGAAGTGCAAAGTCTCAGACAT-3'
	Reverse 5'-GTCAGTCATCTTGTATTGTTGGGCT-3'
mouse-CCR7	Forward 5'-AGGGATGACGTCACCTACAGCCTG-3'
	Reverse 5'-AGGGCAGCCCAAGTCCTTGAAGAG-3'
mouse-TGF-β	Forward 5'-CAACCAACACCCTGAACCCAGAG-3'
	Reverse 5'-CTTCCTTCACCACCATGTTGGACAG-3'
mouse-IL-1β	Forward 5'-TGGATGGAGAGTGTGGATCCCAAG-3'
	Reverse 5'-GGTGGGTGCTGATGTACCAGTTGG-3'
mouse-TNF-α	Forward 5'-CTGACTGAACTTCGGGGTGATCGG-3'
	Reverse 5'-GGCTGGCTTGTCACTCGAATTTTGAGA-3'
mouse-IL-6	Forward 5'-ATAGATAGTCCTTCCTACCCCAATTTCC-3'
	Reverse 5'-GATGGATGAATTGGATGGTCTTGGTCC-3'
mouse-CD206	Forward 5'-AGACAGACGAAATCCCTGCTACTG-3'
	Reverse 5'-CACCCACCCATTCGAAGGCATTC-3'
rat-GAPDH	Forward 5'-ACTCCACGACGTACTCAGCG-3'
	Reverse 5'-GGTCGGAGTCAACGGATTTG-3'
rat-IL-17	Forward 5'-CCATCCATGTGCCTGATGCT-3'
	Reverse 5'-AAGTTATTGGCCTCGGCGTT-3'
rat-RORγt	Forward 5'-AGTGTAATGTGGCCTACTCCT-3'
	Reverse 5'-GCTGCTGTTGCAGTTGTTTCT-3'
rat-IL-21	Forward 5'-TGATGTCTGCCTGTGACCTGTG-3'
	Reverse 5'-CCAGAGGTAGTCAGTGTAGCAGGTG-3'
rat-TGF-β	Forward 5'-CTGCTGACCCCCACTGATAC-3'
	Reverse 5'-AGCCCTGTATTCCGTCTCCT-3'
rat-FOXP3	Forward 5'-CCCAGGAAAGACAGCAACC-3'
	Reverse 5'-TTCTCACAACCAGGCCACTTG-3'
rat-IL-10	Forward 5'-GCCAAGCCTTGTCAGAAATGA-3'
	Reverse 5'-TTTCTGGGGCCATGGTTCTCT-3'

Supplementary Figure S1.

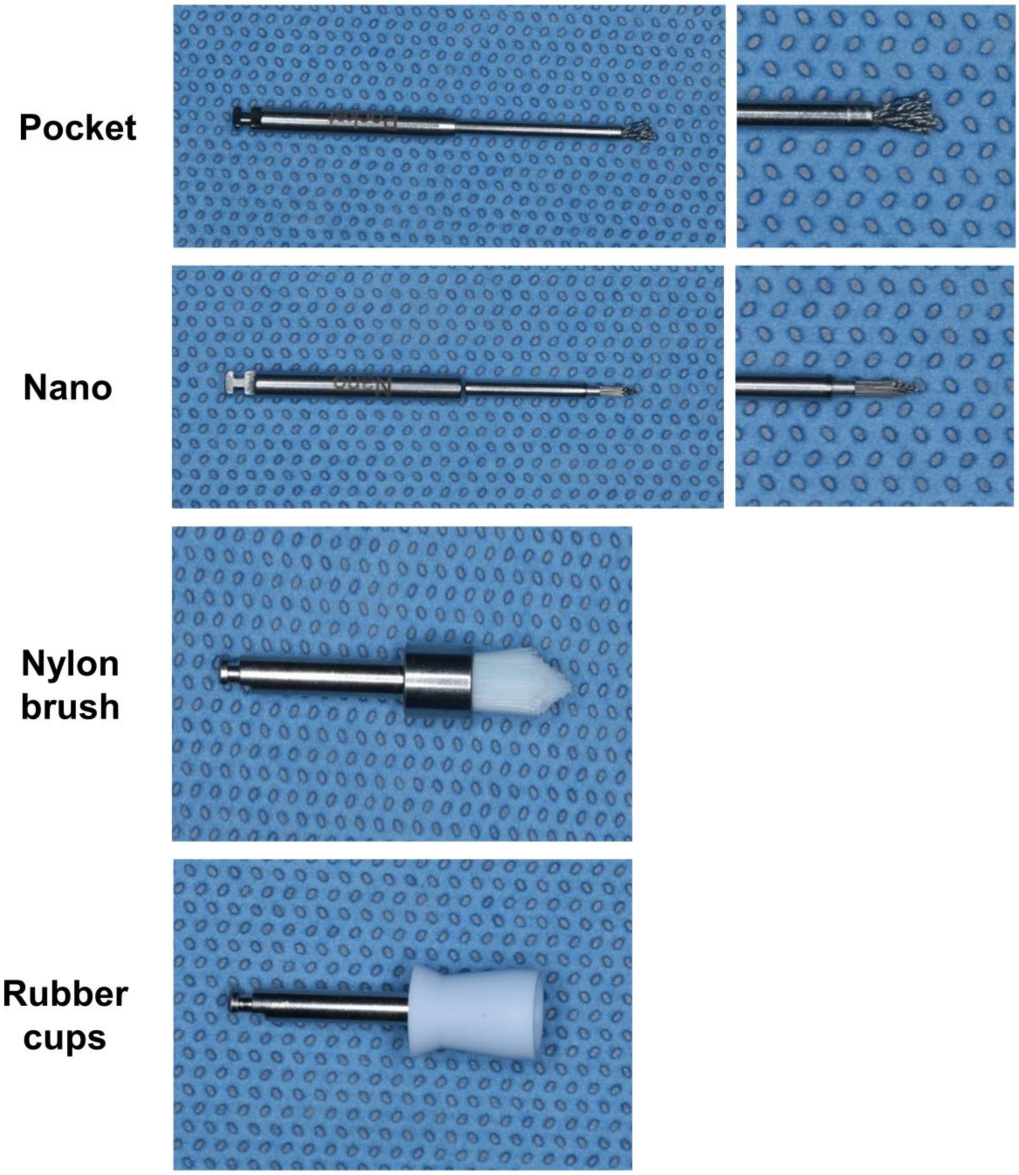

Figure S1. The mechanical debridement tools

Supplementary Figure S2.

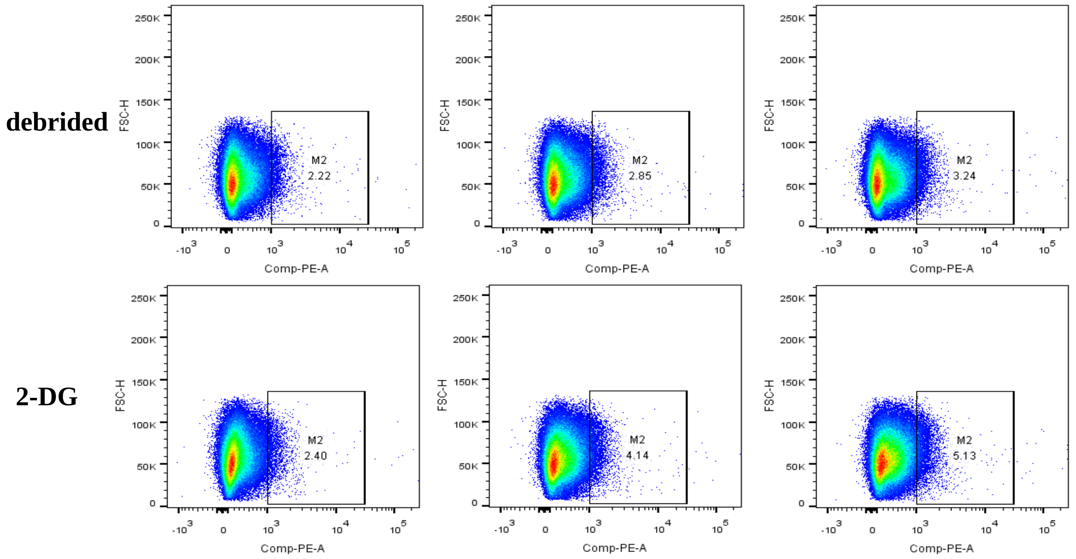

Figure S2. Flow cytometry analysis data: effects of 2-DG on macrophage polarization towards M2 phenotype.
